# Irisin-Driven AMPK-PGC-1α Activation Underlies the Renoprotective Effects of Swimming Exercise in Obesity-Induced Kidney Injury

**DOI:** 10.3390/biom16050727

**Published:** 2026-05-15

**Authors:** Safaa M. Hanafy, Soha S. Zakaria, Mohammad I. Jumaa, Reham A. Al-Dhelaan, Einas M. Yousef

**Affiliations:** 1 Department of Anatomy and Physiology, College of Medicine, Imam Mohammad Ibn Saud Islamic University (IMSIU), Riyadh 13317, Saudi Arabia; aabdelaziz@imamu.edu.sa (S.M.H.); migomaa@imamu.edu.sa (M.I.J.); 2 Department of Biochemistry, College of Medicine, Imam Mohammad Ibn Saud Islamic University (IMSIU), Riyadh 13317, Saudi Arabia; raldhelaan@imamu.edu.sa; 3 Department of Anatomy & Genetics, College of Medicine, Alfaisal University, Riyadh 11533, Saudi Arabia; eyousef@alfaisal.edu

**Keywords:** obesity, exercise, irisin, AMPK, PGC-1α, mitochondrial function, histopathology, renoprotection

## Abstract

*Background:* Obesity often affects kidney health. Irisin, a myokine released during exercise, may exert renoprotective effects. This study examined the effects of swimming-induced irisin on kidney health in obese rats. *Materials and methods:* Sixty male rats were divided into four groups: control non-trained, obese non-trained, control trained, and obese trained. Obesity was induced using a high-fat diet, and an 8-week swimming program was implemented. Measurements included body and kidney weights, renal function markers (serum urea, creatinine, and urinary albumin), lipid profile, fasting glucose, insulin, and HOMA-IR. Levels of skeletal muscle irisin and PGC-1α were measured by ELISA, and citrate synthase activity was assessed spectrophotometrically. Renal tissue analysis included phospho-AMPKα1 (measured by ELISA), Complex I activity, ATP, Malondialdehyde (MDA), superoxide dismutase (SOD) activity (measured spectrophotometrically), and PGC-1α mRNA expression (qRT-PCR). Renal tissues were examined under a light microscope for histopathological evaluation, followed by semi-quantitative scoring of glomerular and tubulointerstitial lesions, morphometric analysis of glomerular tuft area, and a composite score of cleaved caspase-3 immunoexpression. *Results:* Exercise increased skeletal muscle levels of irisin, PGC-1α, and citrate synthase activity. It also activated renal AMPK, improved mitochondrial function, increased PGC-1α mRNA levels, and reduced renal oxidative stress, as evidenced by decreased malondialdehyde (MDA) levels and restored superoxide dismutase (SOD) activity in obese rats. These changes were associated with improved renal function, reduced tubular injury and apoptosis in obese rats, partial restoration of the glomerular tuft area, lower lesion scores, and reduced cleaved caspase-3 immunoexpression. *Conclusions:* These findings suggest that irisin may mediate the renoprotective effects of exercise through the AMPK–PGC-1α pathway, highlighting swimming as a beneficial non-pharmacological intervention and supporting a potential adjunct role for irisin in managing obesity-related CKD.

## 1. Introduction

Obesity is a complex, multifactorial condition characterized by excessive fat accumulation, primarily resulting from poor dietary habits and insufficient physical activity, and is associated with substantial health risks and economic burden [1]. It has been identified as an independent risk factor for the development and progression of chronic kidney disease (CKD), which is associated with metabolic, inflammatory, and hemodynamic disturbances that promote glomerular hyperfiltration, structural injury, and albuminuria [2,3]. The global rise in obesity has been accompanied by a parallel increase in obesity-related CKD [4]. Hence, there is a need for non-pharmacological strategies to address excess adiposity and kidney damage. One of the current best-practice approaches for obesity is increasing physical activity alongside behavior modification, which is now considered to confer organ-protective effects rather than serving solely as a weight-loss intervention [5].

Mitochondrial dysfunction is a key intermediary mechanism linking obesity to renal injury [6]. The kidney is a highly energy-dependent organ; specifically, the proximal tubules rely primarily on mitochondrial oxidative phosphorylation for ATP production [7]. High-fat diet (HFD)-induced obesity alters mitochondrial biogenesis, reduces electron transport chain activity, and impairs ATP production, thereby increasing oxidative stress, tubular damage, and the progression of renal dysfunction [8]. Key indicators of mitochondrial health, such as citrate synthase activity (a marker of mitochondrial content and oxidative capacity) and mitochondrial Complex I (NADH: ubiquinone oxidoreductase) activity, are often reduced in obesity, resulting in impaired electron transport and diminished ATP production in renal cells [9]. Central regulators of mitochondrial homeostasis, including peroxisome proliferator-activated receptor gamma coactivator-1α (PGC-1α) and AMP-activated protein kinase (AMPK), are also dysregulated in obesity, contributing to metabolic inflexibility and increased susceptibility to kidney injury [10].

Irisin is a fibronectin type III domain-containing protein derived from the cleavage of fibronectin type III domain-containing protein 5 (FNDC5). It is a paracrine myokine released into the bloodstream that exerts positive effects on energy expenditure, glucose regulation, and lipid metabolism [11]. Irisin is induced by aerobic exercise and has been shown to activate AMPK signaling, which in turn promotes PGC-1α expression and supports the development of metabolically active tissues [12]. Experimental and clinical evidence shows that low levels of circulating irisin are associated with obesity, insulin resistance, and type 2 diabetes, all of which are strongly linked to CKD risk [11,13]. Administration of exogenous irisin has been shown to lower body weight, enhance metabolic health, and attenuate renal structural damage, suggesting a direct renoprotective effect [11]. Growing evidence supports a bidirectional relationship between physical exercise and irisin-mediated signaling in the kidney. Exercise-induced increases in PGC-1α transcriptional activity in skeletal muscle enhance FNDC5 expression and irisin secretion. Circulating irisin then reaches the kidney, where it binds to integrin αV/β5 receptors on tubular epithelial cells, activating downstream AMPK–PGC-1α signaling and promoting mitochondrial protection [14]. This exercise–irisin–kidney axis is disrupted in obesity, where chronic low-grade inflammation and lipid-induced mitochondrial dysfunction suppress PGC-1α expression and reduce circulating irisin levels, thereby perpetuating progressive renal injury. Understanding how structured exercise training can restore this axis is therefore of considerable clinical relevance in the management of obesity-related CKD [14].

Although there is emerging evidence that disruption of the AMPK–PGC-1α axis contributes to obesity-related mitochondrial dysfunction, and that activation of this pathway may restore mitochondrial integrity and homeostasis [15,16], the effects of structured swimming exercise on this pathway in obesity-associated CKD remain poorly characterized. Additionally, limited information is available regarding its effects on irisin signaling and the integrated assessment of renal function, mitochondrial activity, and histopathology within a single experimental model.

Swimming exercise was selected as the training modality because it is a non-weight-bearing aerobic activity that minimizes mechanical joint stress in obese animals, avoids the confounding effects associated with electrical stimulation used in treadmill protocols, and provides whole-body aerobic engagement in a thermoregulated environment, thereby ensuring metabolic reproducibility and comparability with existing literature [17].

This study aimed to determine whether an 8-week swimming program could attenuate kidney injury induced by an HFD in rats and whether the potential renoprotective effects are reflected in changes in circulating irisin levels, activation of renal AMPK and PGC-1α, and mitochondrial function.

## 2. Materials and Methods

### 2.1. Chemicals

The majority of chemicals, including dithiobis (2-nitrobenzoate) (DTNB), acetyl-CoA, and oxaloacetate (OAA), were obtained from Sigma-Aldrich Chemical Co., St. Louis, MO, USA. All dietary components were obtained from El-Gomhoria Company, Cairo, Egypt.

### 2.2. Animals

#### 2.2.1. The Animals Included in the Experiment

Sixty male albino rats, 6–8 weeks of age and weighing 110–120 g each, were obtained from the animal breeding laboratory of the Faculty of Science, Tanta University, Egypt. The rats were housed in wire-mesh cages at the animal facility of the Faculty of Medicine, Tanta University, with five rats per cage. Environmental conditions were controlled: temperature (22–24 °C), humidity (50–60%), and a 12 h light/dark cycle. The rats had unlimited access to food and water. Cages were cleaned, and bedding was replaced three times a week. Prior to the experiment, animals were acclimated for one week, during which they received standard chow and daily handling to minimize stress. All animals were healthy at study onset and had not undergone any prior procedures or treatments. All procedures involving animals were approved by the Ethics Research Committee of the Faculty of Medicine, Tanta University (Approval No: 31088/02/25) and conducted in accordance with NIH guidelines for the care and use of laboratory animals (NIH Publication No. 85–23, revised in 1996).

#### 2.2.2. Design of the Experiment

**Figure 1:**
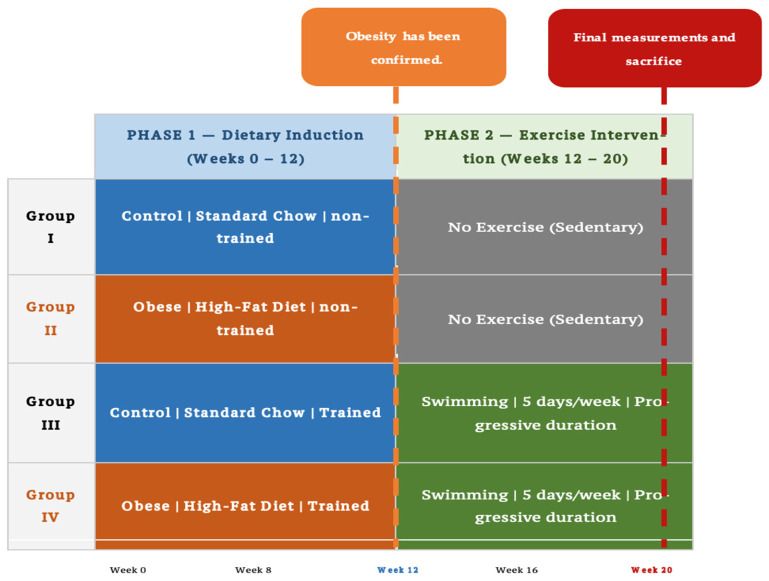
Experimental design and timeline. This study comprised two phases: Phase 1 (Weeks 0–12), dietary induction of obesity via a high-fat diet or standard chow; and Phase 2 (Weeks 12–20), exercise intervention (swimming, 5 days/week, progressive duration) in trained groups. *n* = 15 per group. Color coding: blue indicates standard chow (control diet); orange indicates high-fat diet (obese groups); gray indicates sedentary (no exercise); green indicates swimming exercise intervention.

Rats were randomly assigned to two equal groups of thirty: a control group and an obese group. The control group received a standard caloric diet (59.7% carbohydrates, 10.6% fat, and 27.3% protein) with unrestricted access to water for 12 weeks. The obese group was fed a high-fat diet (27.17% carbohydrates, 53.15% fat, and 19.68% protein) for the same duration to induce obesity. Dietary compositions (g/kg diet) were calculated using an established formula [18]. After 12 weeks, obesity was confirmed in the high-fat diet group when body weight exceeded the mean of the control group by more than 20%. The control group was then subdivided into Group I (control, non-trained) and Group III (control, trained), while the obese group was subdivided into Group II (obese, non-trained) and Group IV (obese, trained) The experimental design is shown in Figure 1.

#### 2.2.3. Exercise Protocol

Swimming exercise was performed five days a week for eight consecutive weeks without additional load in a water tank maintained at thermoneutral temperature (33–35 °C) and a depth of 40–50 cm, allowing unrestricted swimming. Exercise intensity was classified as moderate aerobic, as animals swam continuously without load under thermoneutral conditions. Exercise intensity was monitored by continuous observation: adequate intensity was confirmed when animals maintained active swimming and remained at the water surface throughout the session. Any animal that failed to maintain surface position for >10 consecutive seconds was removed, and the session was terminated. The progressive training protocol was as follows: Week 1, 10 min per session; Week 2, 15 min per session; Weeks 3–8, 30 min per session. This gradual progression minimized acute stress responses and allowed physiological adaptation to exercise [19].

### 2.3. Blood and Tissue Collection

#### 2.3.1. Blood Sampling

At the end of the 20-week study period, rats that underwent overnight fasting were weighed, anesthetized with ketamine, and euthanized. Blood samples were collected in clean, sterile centrifuge tubes and allowed to clot at room temperature for 30 min. Samples were then centrifuged at 1000× *g* for 20 min at 4 °C to obtain serum, which was collected and stored at −80 °C for subsequent biochemical analysis.

#### 2.3.2. Kidney Sampling

Kidneys were removed, weighed, and perfused in situ with ice-cold saline. They were blotted dry and cut into small pieces. One portion was fixed in 10% neutral-buffered formalin for histopathological examination, while the remaining portions were stored at −80 °C for RNA extraction and tissue homogenate preparation.

- Preparation of renal tissue homogenate

Tissue samples were weighed and homogenized in 10 volumes of ice-cold 50 mM phosphate-buffered saline (PBS), pH 7.4, containing 1.15% KCl, using a Potter–Elvehjem tissue homogenizer. Homogenates were centrifuged at 10,000× *g* for 20 min at 4 °C to remove cellular debris. The supernatant was collected and stored at −80 °C for subsequent analysis.

#### 2.3.3. Muscle Tissue Sampling

After anesthesia and decapitation, the soleus and gastrocnemius muscles were carefully dissected, washed, weighed, blotted, and cut into small pieces. Samples were stored at −80 °C for preparation of tissue homogenates, nuclear extracts, and mitochondrial fractions.

- Preparation of muscle tissue homogenate

Muscle tissue was weighed and homogenized in 10 mM cold potassium phosphate-buffered saline, pH 7.4., using a Potter–Elvehjem tissue homogenizer. The crude homogenate was centrifuged at 10,000× *g* for 20 min at 4 °C, and the supernatant was stored at −80 °C for protein analysis and irisin measurement.

- Preparation of nuclear extract

Muscle nuclear extracts for estimating PGC-1α analysis were prepared using a nuclear extraction kit (Catalog No. OP-0022; Epigentek, Farmingdale, NY, USA) according to the manufacturer’s instructions.

- Separation of mitochondrial fraction

A portion of muscle or kidney tissue was rinsed with ice-cold saline, finely minced, and incubated in 10 mM EDTA with 0.05% trypsin for 15 min. Samples were then centrifuged at 800 g for 2 min, and the pellet was homogenized (1:5 *w/v* in ice-cold isolation buffer (300 mM sucrose, 10 mM Tris-HCl, 10 mM KCl, 10 mM EDTA, and 0.5% BSA; pH 7.4) using a tissue homogenizer. The homogenate was centrifuged at 2000 g for 2 min at 4 °C, and the supernatant was subsequently centrifuged at 10,000× *g* for 5 min. The resulting pellet was washed once in BSA-free isolation buffer and centrifuged again at 10,000× *g* for 5 min. The final mitochondrial pellet was resuspended in buffer suitable for protein and enzyme assays. All steps were performed at 4 °C [20].

### 2.4. Biochemical Analysis

All ELISA-based measurements were performed in duplicate for each biological sample. The intra-assay coefficient of variation (CV%) was <8% and the inter-assay CV% was <12% for all assays, both within acceptable limits (<15%). All standard curves exhibited excellent linearity with R^2^ > 0.99. Positive and negative kit controls yielded values within the manufacturer-specified ranges in all assays runs.

#### 2.4.1. Evaluation of Renal Function

- Blood urea and serum creatinine levels were measured using enzymatic–colorimetric methods with commercially available kits (Biodiagnostic, Dokki, Giza, Egypt).
- For assessment of urinary albumin, rats were placed in metabolic cages for 24 h with free access to food and water. Urine samples were centrifuged at 2000× *g* for 10 min at 4 °C. Urinary albumin levels were measured using commercial kits (Biodiagnostic, Dokki, Giza, Egypt). The 24 h urinary albumin excretion rate (UAER) was calculated using the following formula: UAER (μg/24 h) = urinary albumin (μg/mL) × 24 h urine volume (mL) [21].

#### 2.4.2. Assessment of Lipid Profile, Plasma Glucose, Insulin Levels, and HOMA-IR Index

The lipid profile, comprising total cholesterol (TC), triglycerides (TG), high-density lipoprotein cholesterol (HDL-C), and free fatty acids (FFA), was assessed using colorimetric techniques with commercial kits (Biodiagnostic, Dokki, Giza, Egypt). Low-density lipoprotein cholesterol (LDL-C) was calculated using the Friedewald equation [22]. Fasting plasma glucose was determined using the glucose oxidase method (Biodiagnostic, Egypt). Plasma insulin concentrations were measured using a sandwich ELISA kit (SunRed Biological Technology Co., Ltd., Shanghai, China). Insulin resistance was calculated using the following equation [23]:

HOMA-IR = [Fasting insulin (µIU/mL) × Fasting glucose (mg/dL)]/405

#### 2.4.3. Measurement of Irisin in Muscular Tissue Homogenate

Irisin levels in muscle tissue homogenates were quantified using an enzyme-linked immunosorbent assay (ELISA) kit (SunRed Biological Technology Co., Ltd., Shanghai, China).

#### 2.4.4. Measurement of PGC-1α Levels in Muscle Tissue Nuclear Extracts

The concentrations of PGC-1α in the nuclear extract of muscle tissue were quantified using an enzyme-linked immunosorbent assay (ELISA) kit (SunRed Biological Technology Co., Ltd., Shanghai, China).

#### 2.4.5. Citrate Synthase Activity

Citrate synthase activity in muscle mitochondrial fractions was evaluated following three freeze–thaw cycles. Samples were incubated with 0.5 mM oxaloacetate, 0.31 mM acetyl-CoA, and 0.1 mM DTNB in 100 mM Tris-HCl buffer (pH 8.0) at 37 °C for 3 min. Enzyme activity was determined by measuring the decrease in DTNB absorbance at 412 nm using spectrophotometry and expressed as Units/min/mg protein [24].

#### 2.4.6. Adenosine Monophosphate Protein Kinase (AMPK) Levels

Activated AMPK-α1 levels in kidney tissue were measured using a sandwich ELISA specific for phosphorylated AMPK-α1 (T174) (R&D Systems, Minneapolis, MN, USA).

#### 2.4.7. Assessment of Renal Mitochondrial Function

- Complex I (NADH–ubiquinone oxidoreductase) activity in renal mitochondrial fractions was measured spectrophotometrically by monitoring NADH oxidation at 340 nm [25] and expressed as nanomoles of NADH oxidized per minute per milligram of protein.
- ATP concentration was measured using a commercial assay kit (Cat. No. S0027, Beyotime Biotechnology, Haimen, Jiangsu, China) in accordance with the manufacturer’s instructions.

#### 2.4.8. Measurement of PGC-1α mRNA Expression

PGC-1α mRNA expression in renal tissue was assessed by qRT-PCR according to the manufacturer’s instructions [26]. Total RNA was extracted from renal tissue homogenates using RNeasy Purification Reagent (Qiagen, Valencia, CA, USA). RNA integrity was assessed by gel electrophoresis. First-strand cDNA was synthesized from 4 μg total RNA using oligo(dT)12-18 primer and SuperScript™ II RNase Reverse Transcriptase, with incubation at 42 °C for 1 h. The kit was obtained from the SuperScript^®^ Choice System (Life Technologies, Breda, The Netherlands). Real-time PCR (RT–PCR) was performed using SYBR Green PCR Master Mix (Applied Biosystems, Foster City, CA, USA) under the following conditions: 10 min of denaturation at 95 °C, followed by 40 cycles of 15 s at 95 °C and 60 s at 60 °C (annealing/extension). Relative gene expression was calculated using the ΔΔCt approach, with β-actin as the internal control. Primer sequences were as follows: PGC-1α (NM_031347.1): Forward 5′-ACATCGCAATTCTCCCTT-3′, Reverse 5′-TCTTGAGCCTTTCGTGCTC-3′; β-actin (XM_017604191.1): Forward 5’-CCAGGCTGGATTGCAGTT-3’, Reverse 5’-GATCACGAGGTCAGGAGATG-3’.

#### 2.4.9. Renal Redox Status

Malondialdehyde (MDA), a marker of lipid peroxidation, was quantified in renal tissue homogenates by the thiobarbituric acid (TBA) reactive substance method, with absorbance read at 532 nm and expressed as nmol/mg protein [27]. Superoxide dismutase (SOD) activity was assayed spectrophotometrically and expressed as U/mg protein [28].

#### 2.4.10. Total Protein Concentration

Total protein concentrations were measured in muscle and kidney homogenates, nuclear extracts, and mitochondrial fractions [29].

### 2.5. Histological and Morphometric Analyses

For paraffin embedding, small portions of renal cortex were fixed in 10% neutral-buffered formalin, dehydrated, cleared, and embedded in paraffin. Sections (5 μm) were cut using a microtome and mounted on glass slides. Tissue sections were stained with hematoxylin and eosin (H&E) and examined under a light microscope for histological evaluation [30]. Slides were assessed using an Olympus light microscope (Leica, Switzerland).

For semi-quantitative histological assessment, six randomly selected, non-overlapping fields per section (×400) were examined by two independent histopathologists. Parameters assessed included renal corpuscles (glomerulosclerosis, glomerular necrosis, and Bowman’s space dilation); renal tubules (dilatation, coagulative necrosis, cloudy swelling, and lipid droplet accumulation); and interstitial tissue (vascular congestion and inflammatory cell infiltration) [31]. Lesions were graded on a scale from 0 to 3 (0 = no changes, 1 = mild, 2 = moderate, and 3 = severe). Mean scores were calculated and further categorized as “–“ (no changes), 1+ (mild), 2+ (moderate), and 3+ (severe histopathological damage).

For morphometric analysis, the glomerular tuft area (µm^2^) was measured using ImageJ (version 1.53K, National Institutes of Health, Bethesda, MD, USA) after calibration with the scale bar. Measurements were performed on six non-overlapping fields at ×400 magnification from H&E-stained sections (six animals/group).

### 2.6. Immunohistochemical Analysis

Immunohistochemical staining was performed as previously described [32,33]. Paraffin sections (5 μm thick) were cut and mounted on charged glass slides, rehydrated, and deparaffinized. Endogenous peroxidase activity was blocked using 10% hydrogen peroxide for ten minutes. For antigen retrieval, sections were microwaved in 0.01 mol/L citrate buffer (pH 6) for 5 min. Slides were washed in PBS (pH 7.4) to minimize nonspecific staining and incubated with 1% BSA in PBS for 30 min at 37 °C. Except for negative controls, sections were incubated overnight at room temperature with rabbit polyclonal anti-cleaved caspase-3 antibody (catalog number GB115733-100, Servicebio, Wuhan, China; 1:500 dilution, specific for cleaved caspase-3). After washing with PBS for 10 min, slides were incubated with biotinylated goat secondary antibody for 15 min. Color development was achieved using DAB, followed by Mayer’s hematoxylin counterstaining, dehydration, and mounting. Cleaved caspase-3 immunoreactivity appeared as brown cytoplasmic and/or nuclear staining.

Immunohistochemical expression of caspase-3 was evaluated in ten randomly selected, non-overlapping high-power fields (×400) per slide by assessing both the percentage of positively stained cells and staining intensity. The percentage score was graded as 0 (0%), 1 (1–10%), 2 (11–30%), 3 (31–50%), 4 (51–80%), or 5 (>80%), while staining intensity was graded as 1 (weak), 2 (moderate), or 3 (strong). The two scores were summed to yield a final immunoreactivity score ranging from 0 to 8 [34]. The mean score per animal was calculated from the ten examined fields and used for statistical analysis.

### 2.7. Statistical Analysis

Statistical analysis was performed using R software (version 4.3). Data distribution normality was assessed using the Shapiro–Wilk test for each group, and homogeneity of variances was evaluated using Levene’s test. Based on these assumptions, an adaptive analytical strategy was applied. Parameters meeting both normality and homogeneity of variance assumptions were compared across the four groups using one-way analysis of variance (ANOVA) followed by Tukey’s HSD post hoc test. Parameters with normal distributions but unequal variances were analyzed using Welch’s ANOVA followed by the Games–Howell post hoc test. Parameters violating the normality assumption were analyzed using the Kruskal–Wallis test followed by Dunn’s post hoc test with Bonferroni adjustment. Normally distributed continuous variables are presented as mean ± standard deviation, whereas non-normally distributed variables are presented as median [interquartile range]. The level of statistical significance was set at *p* < 0.05, and all tests were two-tailed. Histopathological scoring data were analyzed across experimental groups, with each value derived from six non-overlapping high-power fields per section. Morphometric data (glomerular tuft area, µm^2^) were analyzed by calculating the mean per animal across multiple glomeruli, and these individual means were used for statistical comparisons.

## 3. Results

### 3.1. Body Weight and Kidney Weight

**Figure 2:**
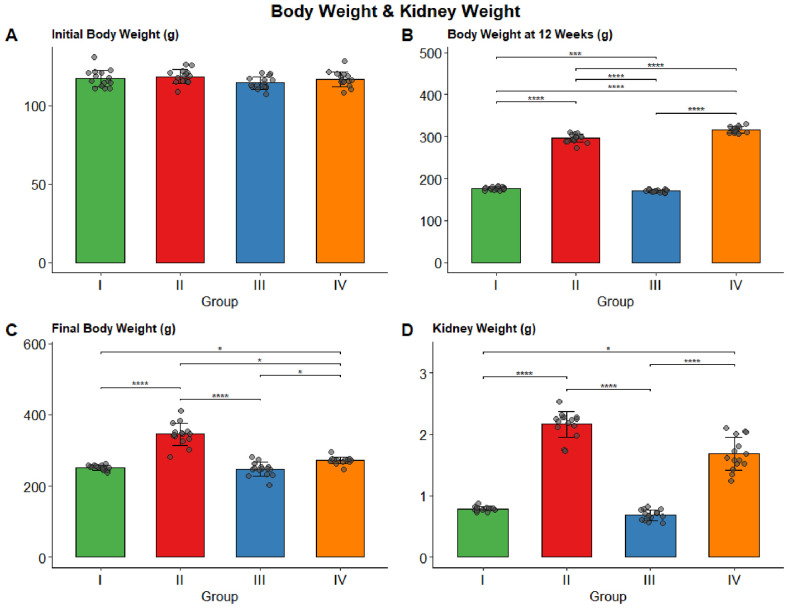
Body weight (g) and kidney weight (g) across the four study groups. Bars represent mean values, with error bars indicating standard deviation. Individual data points are overlaid as jittered dots. Significance brackets indicate pairwise comparisons from Dunn’s or Tukey’s post hoc tests (* *p* < 0.05, *** *p* < 0.001,**** *p* < 0.0001). (**A**) Initial body weight (g); (**B**) body weight at 12 weeks (g); (**C**) final body weight (g); (**D**) kidney weight (g).

No significant difference in initial body weight was observed among the four groups (F = 1.973, *p* = 0.129), confirming successful randomization at baseline. After 12 weeks of high-fat feeding, and before the exercise intervention, body weight was markedly higher in the obese groups (Groups II and IV) than in the lean controls (Groups I and III), with all pairwise comparisons statistically significant (Welch’s ANOVA, F = 2121.65, *p* < 0.001). At the end of this study, Groups II and IV had significantly higher final body weight than Groups I and III (*p* < 0.05). Importantly, swimming exercise training attenuated weight gain in obese rats, with Group IV showing a significantly lower final body weight than Group II (*p* = 0.033). Training did not affect body weight in lean controls (Group I vs. Group III, *p* = 1.000). Kidney weight followed a similar pattern, with Group II showing the highest median value of 2.24 g [2.13, 2.27], which was significantly higher than that of all other groups except Group IV. Swimming exercise training significantly reduced kidney weight in Group IV compared with Group III (*p* < 0.001). Full data are presented in Table 1 and Figure 2.

### 3.2. Renal Function, Lipid Profile, and Glycemic Parameters

**Figure 3:**
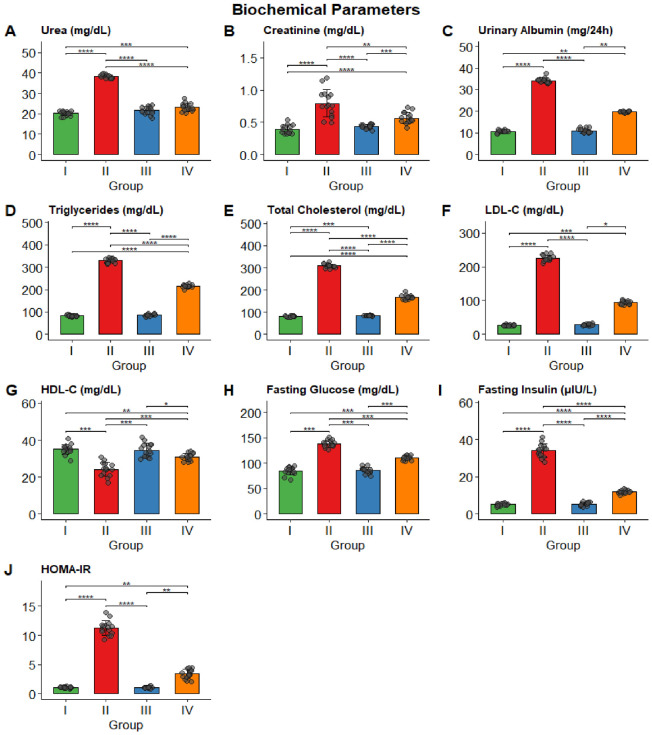
Biochemical parameters across the four study groups, (**A**) Urea (mg/dL); (**B**) creatinine (mg/dL); (**C**) urinary albumin (mg/24 h); (**D**) triglycerides (mg/dL); (**E**) total cholesterol (mg/dL); (**F**) LDL-C (mg/dL); (**G**) HDL-C (mg/dL); (**H**) fasting glucose (mg/dL); (**I**) fasting insulin (µIU/L); (**J**) HOMA-IR. Bars represent mean or median values, with error bars indicating SD. Individual data points are overlaid. (* *p* < 0.05, ** *p* < 0.01, *** *p* < 0.001, **** *p* < 0.0001).

Group II (obese non-trained) exhibited significantly higher serum urea (38.31 ± 0.87 mg/dL), creatinine (0.79 ± 0.21 mg/dL), and urinary albumin (median 34.27 mg/24 h) than all other groups (all *p* < 0.001). Lipid parameters were markedly altered in Group II, with triglycerides exceeding 329 mg/dL, total cholesterol exceeding 309 mg/dL, and LDL-C at a median of 224.60 mg/dL. HDL-C was significantly lower in Group II (24.16 ± 3.78 mg/dL) than in Groups I and III. Fasting glucose, insulin, and HOMA-IR were also significantly elevated in Group II. Swimming exercise training (Group IV) attenuated these alterations, with significant improvements compared with Group II across all measured parameters. Group III (control trained) did not differ significantly from Group I for most parameters. The complete results are presented in Table 2 and Figure 3.

### 3.3. Irisin, PGC-1α Protein, and Citrate Synthase Activity in Muscle Tissue

**Figure 4:**
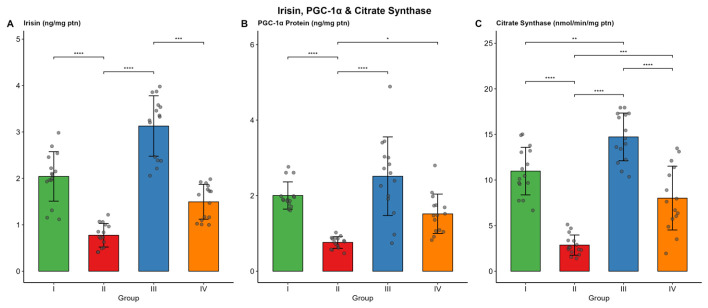
Irisin levels, PGC-1α protein, and citrate synthase activity in muscle tissue across the four study groups. Bars represent mean or median values, with error bars indicating SD. Individual data points are overlaid. (* *p* < 0.05, ** *p* < 0.01, *** *p* < 0.001, **** *p* < 0.0001). (**A**) Irisin (ng/mg ptn); (**B**) PGC-1α protein (ng/mg ptn); (**C**) citrate synthase (nmol/min/mg ptn).

Irisin levels were significantly lower in Group II (median 0.74 ng/mg ptn) than in Group I (2.08) and Group III (3.33) (both *p* < 0.001). Group III (control trained) exhibited the highest irisin levels, which were also significantly higher than those in Group IV (*p* < 0.001). PGC-1α protein followed a similar pattern, with Group II showing the lowest median (0.78 ng/mg ptn). Citrate synthase activity was highest in Group III (14.73 ± 2.62 nmol/min/mg ptn) and lowest in Group II (2.86 ± 1.11). Exercise training increased citrate synthase activity in Group IV compared with Group II (*p* < 0.001). The results are presented in Table 3 and Figure 4

### 3.4. Renal Mitochondrial Function Markers, PGC-1α mRNA Expression, and Oxidative Stress Markers

Phospho-AMPKα1 levels were significantly lower in Group II (median 0.06 pg/µg protein) than in Groups I and III (both *p* < 0.001). Group III (control trained) exhibited significantly higher phospho-AMPKα1 levels than Group IV (*p* < 0.001). Complex I activity was lowest in Group II (38.39 ± 16.32 µmol NADH/min/mg protein) and highest in Group III (106.07 ± 8.39). ATP levels followed the same trend, with Group II at 0.52 ± 0.08 and Group III at 1.15 ± 0.11 µmol/mg protein. Renal PGC-1α mRNA expression was markedly reduced in Group II (median 0.69) compared with Group I (1.95, *p* < 0.001). Exercise training partially restored expression in Group IV (median 1.04), although values remained significantly lower than those in Group I (*p* = 0.024) and Group III (*p* < 0.001). 

**Figure 5:**
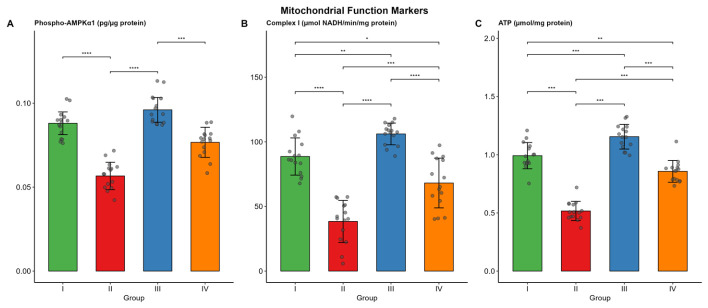
Renal mitochondrial function markers (phospho-AMPKα1, Complex I activity, and ATP levels) across the four study groups. Bars represent mean or median values, with error bars indicating SD. Individual data points are overlaid. (* *p* < 0.05, ** *p* < 0.01, *** *p* < 0.001, **** *p* < 0.0001). (**A**) Phospho-AMPKα1 (pg/µg protein); (**B**) Complex I (µmol NADH/min/mg protein); (**C**) ATP (µmol/mg protein).

**Figure 6:**
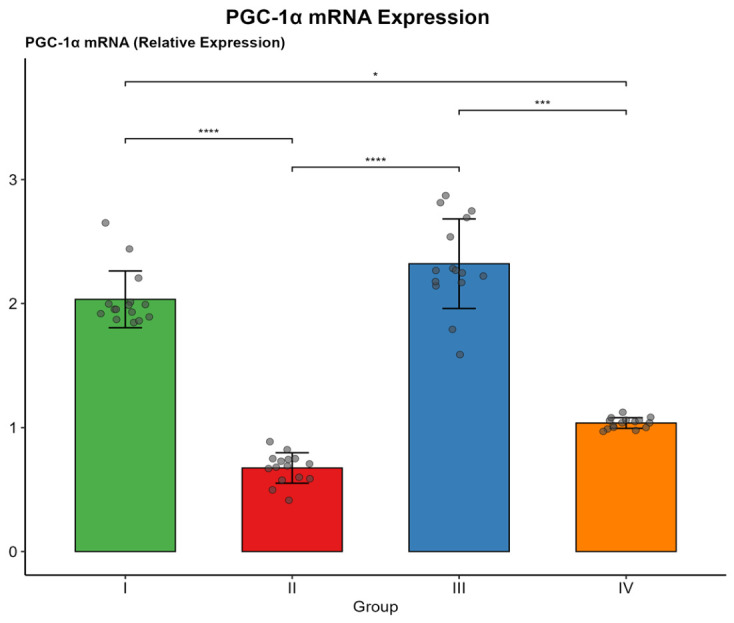
Renal PGC-1α mRNA expression (relative expression) across the four study groups. Bars represent median values, with error bars indicating SD. Individual data points are overlaid. (* *p* < 0.05, *** *p* < 0.001, **** *p* < 0.0001).

Renal MDA levels were significantly higher in Group II than in Groups I and III (both *p* < 0.001), indicating marked lipid peroxidation. Group IV had significantly lower MDA levels than Group II (*p* < 0.001), though it remained significantly above control values. SOD activity showed an inverse trend, with the lowest in Group II and the highest in Group I (*p* < 0.001). Group III showed comparable preservation. Group IV exhibited significantly higher values than Group II (*p* < 0.001) but significantly lower values than Groups I and III.The results are presented in Table 4 and Figure 5 and Figure 6.

### 3.5. Histopathological, Morphometric, and Immunohistochemical Results

#### 3.5.1. Histopathological and Morphometric Results

**Figure 7:**
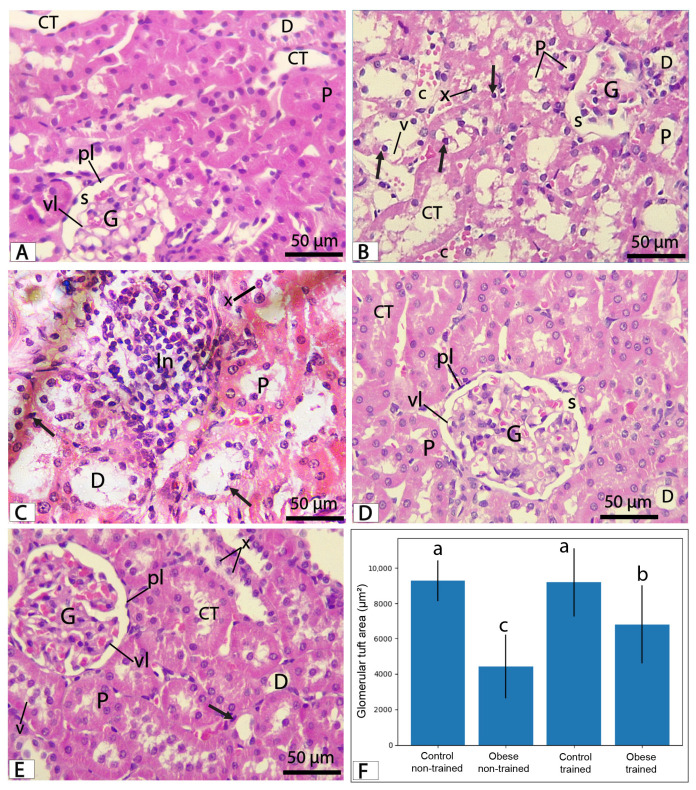
Photomicrographs of H&E-stained kidney cortical sections from different experimental groups. (**A**) Control non-trained group showing a renal corpuscle with a well-defined glomerulus (G) surrounded by Bowman’s capsule, with a normal Bowman’s space (s). Bowman’s capsule has a visceral layer (vl) covering the glomerular capillaries and a parietal layer (pl) lined by simple squamous epithelium. The proximal convoluted tubules (P) exhibit narrow lumina and are lined by cuboidal epithelial cells with a brush border and eosinophilic cytoplasm, whereas distal convoluted tubules (D) display wider lumina and less eosinophilic cytoplasm. Collecting tubules (CT) are lined by simple cuboidal epithelium with distinct cell boundaries. (**B**,**C**) Obese non-trained group showing widening of Bowman’s space (s). Renal tubules, including proximal (P) and distal (D) convoluted tubules, display epithelial degeneration characterized by cytoplasmic vacuolization (V), pyknotic nuclei (arrows), and intraluminal exfoliated cells (x). Collecting tubules (CT) appear dilated with a disrupted epithelial lining. Prominent interstitial inflammatory cell infiltration (In) and congested blood capillaries (c) are evident between the tubules. (**D**) Control trained group showing histological features similar to the control non-trained group, including intact renal corpuscle (G, s, vl, pl) and tubules (P, D, CT). (**E**) Obese trained group showing marked improvement in renal histological architecture, with a relatively preserved renal corpuscle exhibiting a near-normal Bowman’s space (s) and mostly intact tubules, including proximal (P), distal (D), and collecting tubules (CT), with mild residual alterations, such as cytoplasmic vacuolization (V), occasional pyknotic nuclei (arrows), and limited intraluminal exfoliated cells (X). (**F**) Glomerular tuft area (µm^2^) across experimental groups. Data are presented as mean ± SD. Bars with different letters indicate statistically significant differences (*p* < 0.05). (H&E, ×400 magnification). Scale bar = 50 µm.

Histological examination of H&E-stained kidney sections revealed normal renal architecture in the control non-trained group, with a well-organized cortex composed of renal corpuscles and tubules. The Malpighian corpuscles consisted of glomeruli surrounded by Bowman’s capsule. Renal tubules include proximal convoluted tubules, which are lined by tall cuboidal cells with clear brush borders, acidophilic cytoplasm, and narrow lumens, while distal convoluted tubules are lined by simple cuboidal epithelium with a less prominent brush border and wider lumens. Collecting tubules are lined by simple cuboidal epithelial cells (Figure 7A). In contrast, the obese non-trained group demonstrated marked histopathological alterations, including widening of Bowman’s space, exfoliation of tubular epithelium, cytoplasmic vacuolation, and pyknotic nuclei. Disruption of the brush border in some proximal tubules and evident lymphocytic infiltration in the interstitium were also observed. Semi-quantitative scoring confirmed that cloudy swelling and inflammatory infiltration were the most prominent lesions in this group, predominantly corresponding to moderate-to-severe grades (Figure 7B,C, and Table 5).

The control trained group exhibited histological architecture comparable to that of the control non-trained group, with no detectable alterations on scoring (Figure 7D). The obese trained group demonstrated marked improvement in histological architecture, with renal corpuscles and most renal tubules appearing improved compared with the obese non-trained group. However, mild changes persisted, including occasional tubular epithelial cells with pyknotic nuclei, cytoplasmic vacuolization, and limited intraluminal exfoliation (Figure 7E). These findings were supported by semi-quantitative scoring, which indicated a significant reduction in the severity of histological alterations compared with the non-trained groups (Table 5).

Morphometric analysis revealed a significant reduction in the glomerular tuft area in the obese non-trained group compared with the control groups (*p* < 0.001). Exercise training significantly increased the glomerular tuft area in the obese trained group compared with the obese non-trained group (*p* < 0.05), although values remained significantly lower than those of the control groups. No significant difference was observed between the control non-trained and control trained groups (Figure 7F).

#### 3.5.2. Immunohistochemical Results

**Figure 8:**
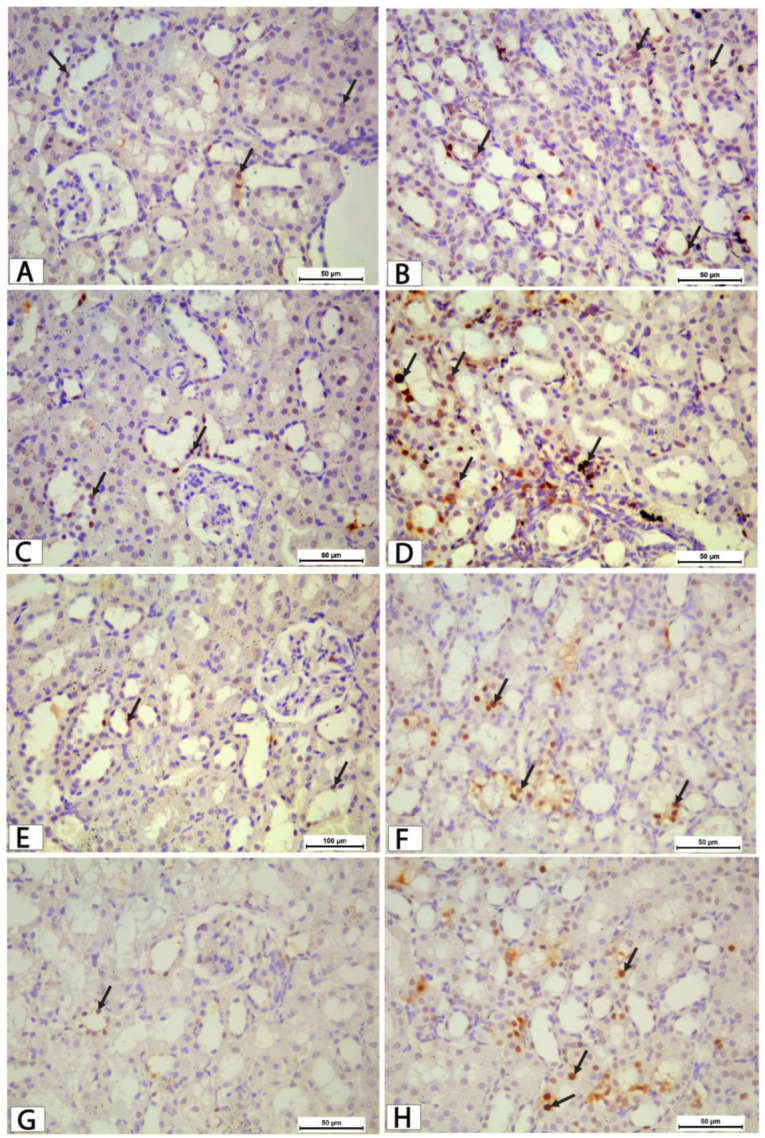
Photomicrographs of kidney sections immunostained for cleaved caspase-3 in the different experimental groups. (**A**,**B**) Control non-trained group showing minimal cleaved caspase-3 immunoexpression in a few positively stained tubular epithelial nuclei (arrows). (**C**,**D**) The obese non-trained group showed a marked increase in cleaved caspase-3 expression in renal tubular epithelial cells and glomerular cells (arrows), indicating enhanced apoptotic activity. (**E**,**F**) The control trained group showing low-to-moderate cleaved caspase-3 immunoreactivity, comparable to that of the control non-trained group, with few positively stained tubular epithelial cells (arrows). (**G**,**H**) The obese trained group showed reduced cleaved caspase-3 immunoexpression compared with the obese non-trained group(arrows), suggesting attenuation of apoptosis. Scale bar = 50 µm.

Immunohistochemical examination of kidney sections revealed minimal cleaved caspase-3 immunoreactivity in the control non-trained group, with only a few positively stained nuclei observed in renal tubular cells, while glomeruli exhibited no immunoreactivity (Figure 8A,B). In contrast, the obese non-trained group exhibited a marked increase in caspase-3 expression, predominantly in renal tubular epithelial cells and, to a lesser extent, within glomerular structures (Figure 8C,D). This increase was accompanied by evident tubular degenerative changes, including cytoplasmic vacuolation, indicating enhanced apoptotic activity in renal tissue.

The control trained group showed low-to-moderate cleaved caspase-3 expression comparable to that of the control non-trained group, with limited positively stained cells (Figure 8E,F). Notably, the obese trained group showed a noticeable reduction in cleaved caspase-3 expression compared with the obese non-trained group, as evidenced by fewer positively stained nuclei in both tubules and glomeruli (Figure 8G,H). However, the level of immunostaining in this group remained higher than that of the control groups, suggesting partial attenuation of obesity-induced renal apoptosis following exercise training.

**Figure 9:**
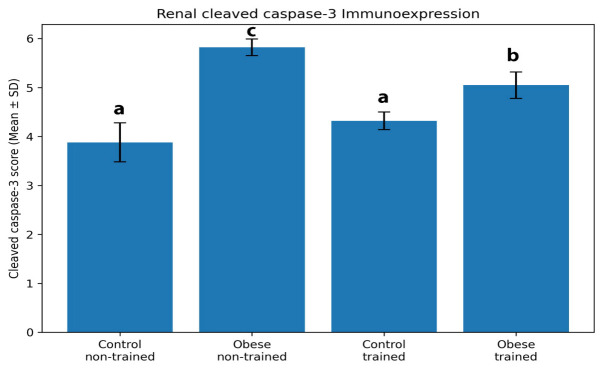
Renal cleaved caspase-3 immunoexpression in the different experimental groups. Data are presented as mean ± SD. Different letters above the bars indicate statistically significant differences between groups at *p* < 0.05.

Semi-quantitative analysis (Figure 9) confirmed these histological findings, as one-way ANOVA revealed a highly significant difference among the experimental groups (F (3,20) = 59.33, *p* < 0.001). Post hoc Tukey analysis demonstrated a significant increase in cleaved caspase-3 expression in the obese non-trained group compared with both control groups (*p* < 0.001). The obese trained group showed a significant reduction in cleaved caspase-3 expression compared with the obese non-trained group (*p* = 0.0004), although values remained significantly higher than those in the control groups. No significant difference was detected between the control non-trained and control trained groups (*p* = 0.052).

## 4. Discussion

Epidemiological data indicate that obesity affects more than one billion people worldwide and is projected to continue rising, with overweight and obese populations facing a two- to sevenfold increased risk of end-stage renal disease compared with normal-weight counterparts [35,36]. The public health burden is compounded by the frequent coexistence of obesity-related CKD with cardiovascular disease, type 2 diabetes, and hypertension, forming a cluster of comorbidities that amplifies morbidity, mortality, and healthcare costs [36]. In this context, structured exercise programs have gained increasing recognition as cost-effective, non-pharmacological interventions that confer organ-protective benefits beyond weight reduction, including improvements in endothelial function, systemic inflammation, and cardiorenal outcomes [37]. In this study, HFD feeding caused a significant and progressive increase in both body and kidney weight in the obese groups compared with the controls, confirming dietary induction of an obese phenotype. These results align with reports that HFD-induced obesity leads to ectopic renal lipid deposition, which drives glomerular hyperfiltration, tubular cell hypertrophy, and low-grade inflammatory infiltration [38]. Histopathological analysis of Group II supported these findings, showing increased Bowman’s space width, disruption of the proximal tubular brush border, cytoplasmic vacuolization, intraluminal cellular exfoliation, deeply stained nuclei, and lymphocytic infiltration in renal tissue. The notable reduction in body and kidney weight in the obese trained group (Group IV) after swimming exercise highlights the well-known metabolic benefits of aerobic training, including enhanced lipid oxidation, increased skeletal muscle glucose uptake, and upregulation of thermogenic pathways [37]. Similarly, histological examination of Group IV showed near normalization of the renal cortical structure, with only occasional tubular vacuolation and rare intraluminal exfoliation, demonstrating exercise’s role in alleviating renal hypertrophy [39].

Elevated levels of urea, creatinine, and albumin in the urine observed in Group II indicate significant glomerular and tubular damage, a condition often linked to obesity-related nephropathy. Obesity-related hyperfiltration damages podocytes and weakens the glomerular filtration barrier, while lipid accumulation within tubular epithelial cells hampers proximal tubular reabsorption, leading to increased albuminuria [40]. These functional impairments were confirmed by cleaved caspase-3 immunohistochemistry, which revealed strong cytoplasmic positivity in tubular epithelium and glomerular capillary tuft cells in Group II. Additionally, exfoliated, degenerating epithelial cells were observed within tubular lumina, indicating substantial apoptotic cell death that contributes to kidney injury mechanisms [41].

Swimming exercise in Group IV significantly improved all kidney function markers, consistent with research showing that aerobic activity lowers glomerular pressure, reduces podocyte apoptosis, and restores tubular transport by reducing oxidative stress and inflammation [42]. These functional improvements were supported by the histopathological and morphometric findings. The obese trained group showed partial restoration of the glomerular tuft area, reduced glomerulosclerosis, narrower Bowman’s space dilation, and lower tubular and interstitial lesion scores compared with the obese non-trained group. Preservation of the glomerular structure, along with reduced tubular epithelial injury, may explain the lower urinary protein and albumin excretion observed after exercise. Furthermore, this improvement was evidenced by markedly lower cleaved caspase-3 immunoreactivity in Group IV, where only scattered tubular epithelial cells and occasional glomerular capillary tuft cells showed weak positivity, indicating a substantial decrease in renal cell death.

The dyslipidemia observed in Group II, marked by increased triglycerides, total cholesterol, and LDL-C, along with decreased HDL-C, is a well-known contributor to renal lipotoxicity. Excess circulating lipids undergo oxidative changes, producing reactive lipid species such as oxidized LDL and ceramides, which activate NF-κB signaling in mesangial and tubular cells, sustaining a pro-inflammatory and pro-fibrotic renal environment [38]. Histology in Group II revealed mesangial widening, vacuolated tubular epithelium containing lipid droplets, and periglomerular fibrosis, consistent with early obesity-related nephropathy. Swimming exercise in Group IV normalized lipid profile, likely through increased lipoprotein lipase activity and improved hepatic VLDL clearance [43], thereby reducing renal lipotoxic stress.

The significant hyperglycemia, hyperinsulinemia, and insulin resistance (HOMA-IR) in Group II reflect a metabolic syndrome phenotype with renal consequences. Advanced glycation end-products (AGEs) formed during hyperglycemia cross-link glomerular basement membrane proteins, impairing selective permeability, and activate the receptor for AGEs (RAGE), initiating downstream oxidative and inflammatory cascades that promote mesangial expansion and tubulo-interstitial fibrosis [44]. Swimming exercise in Group IV improved insulin sensitivity through increased GLUT4 translocation in skeletal muscle, reduced adipose-derived inflammatory cytokines, and suppressed hepatic gluconeogenesis, collectively alleviating glucotoxic renal injury [45].

The most significant finding of this study is the reduction in skeletal muscle irisin and PGC-1α levels in obese untrained rats, followed by a substantial increase after swimming exercise. Irisin is cleaved from FNDC5 and released from skeletal muscle in response to exercise, while PGC-1α, a key regulator of mitochondrial biogenesis, promotes FNDC5 transcription, linking mitochondrial remodeling to irisin secretion [46]. Obesity-associated reductions in PGC-1α and irisin in obesity result from chronic inflammation and lipid-induced mitochondrial dysfunction, in which ceramide and diacylglycerol activate PP2A and PKCθ, leading to AMPK inactivation and suppression of PGC-1α [47]. These findings support previous reports showing that obesity and lack of physical activity create a cycle of declining irisin levels that impairs mitochondrial biogenesis and perpetuates metabolic dysfunction [48]. Even acute swimming exercise has been shown to activate FNDC5 expression in soleus and gastrocnemius muscles and promote UCP1 expression in subcutaneous adipose tissue, without altering circulating irisin or PGC-1α levels, suggesting that activation of this axis is modality- and fiber-type-dependent [49].

Notably, irisin levels in Group IV, although significantly higher than in Group II, remained lower than in Group III despite equal exercise exposure, suggesting a blunted irisin response in obesity. Clinical data similarly show that structured exercise in obese individuals and patients with type 2 diabetes does not consistently elevate circulating irisin, reflecting attenuated endocrine adaptation [50]. Chronic inflammation and elevated TNF-α and IL-6 inhibit PGC-1α activity, limiting FNDC5/irisin synthesis in skeletal muscle and imposing a metabolic ‘ceiling’ on irisin production [51]. This ceiling effect is further supported by incomplete recovery of renal phospho-AMPKα1, Complex I activity, ATP levels, and PGC-1α mRNA expression in Group IV relative to Group II, indicating persistent metabolic limitation rather than exercise failure [52]. These findings suggest that exercise alone may be insufficient to fully restore renal mitochondrial function in established obesity, and that adjunct strategies such as AMPK activators or anti-inflammatory interventions may be required [53]. Citrate synthase (CS) activity, a marker of mitochondrial density, was reduced in Group II, indicating impaired oxidative capacity [54]. Swimming exercise significantly restored CS activity in Group IV, consistent with PGC-1α/TFAM-mediated mitochondrial biogenesis [55,56]. This restoration supports increased energetic capacity and amplifies irisin-mediated systemic effects [56]. Lu et al. similarly demonstrated that swimming exercise increases serum irisin and reduces adiposity in high-fat-diet-fed rats, with positive correlations between PGC-1α and irisin expression [57]. This study confirmed the role of oxidative damage in obesity-related renal injury, showing increased MDA and decreased SOD in obese, untrained rats. These findings align with reports linking high-fat diet–induced obesity to ROS and mitochondrial dysfunction. Exercise lowered MDA, increased SOD, and up-regulated PGC-1α–driven MnSOD, thereby enhancing superoxide neutralization and protecting mitochondria. These results support the irisin–AMPK–PGC-1α pathway as key to exercise-mediated renal protection. [8,42]

Renal phospho-AMPKα1 was reduced in Group II and restored after exercise, highlighting a mechanistic link between irisin signaling and renal mitochondrial function. AMPK activation reduces mTORC1-driven hypertrophy, inhibits NLRP3 inflammasome activation, suppresses TGF-β/Smad fibrosis signaling, and activates PGC-1αthrough direct phosphorylation at Thr-177 and Ser-538 [58,59]. Irisin activates renal AMPK via integrin αV/β5 receptors on tubular epithelial cells, triggering downstream phosphorylation cascades [60]. The simultaneous reductions in AMPKα1 phosphorylation and ATP levels in the obese kidney reflect bioenergetic failure caused by the combined effects of lipotoxicity, glucotoxicity, and irisin deficiency. The exercise-induced increase in circulating irisin in Group IV thus reactivates renal AMPK activity, providing a clear mechanistic explanation for the observed improvements in renal function [61].

The significant reduction in mitochondrial Complex I (NADH: ubiquinone oxidoreductase) activity in Group II is particularly important, as Complex I is the primary entry point for NADH-derived electrons in the ETC and a key site of ROS production during substrate overload [60]. In obesity, excess reducing equivalents generated by β-oxidation of surplus fatty acids cause mitochondrial membrane hyperpolarization and electron leakage, leading to superoxide formation at Complexes I and III. This condition, known as reductive stress, damages Complex I through oxidative modification of its iron–sulfur clusters and carbonylation of catalytic subunits [62]. This damage impairs TCA cycle activity and creates a metabolic bottleneck, reflected in significantly lower ATP levels in the obese kidney [63]. Although direct histological markers of oxidative stress, such as immunohistochemistry for 4-hydroxynonenal (4-HNE) or 8-hydroxy-2′-deoxyguanosine (8-OHdG), were not assessed in the current study, the biochemical evidence of increased MDA and decreased SOD in the obese, non-trained group is fully consistent with oxidative stress as a central mechanistic driver. The reductions in Complex I activity and ATP levels in Group II are hallmarks of mitochondrial reductive stress caused by excess fatty acid β-oxidation and are consistent with published data showing elevated renal levels of 4-HNE and malondialdehyde in HFD-obese rodents. [59]. Furthermore, oxidative stress is a well-established transcriptional repressor of PGC-1α via NF-κB-dependent mechanisms, thereby accounting for the observed suppression of PGC-1α mRNA in Group II. Swimming exercise restored Complex I activity and ATP levels in Group IV by promoting PGC-1α-driven upregulation of MnSOD, which neutralizes mitochondrial superoxide and protects Complex I from oxidative damage [64]. The significant suppression of renal PGC-1α mRNA in Group II, and its upregulation after exercise, support the transcriptomic evidence underlying the protein-level findings. Obesity-related epigenetic silencing of PGC-1α occurs through DNMT3B-dependent hypermethylation of promoter cytosine residues [65] and post-transcriptional destabilization by miR-23a and miR-696 [66]. Exercise reverses these epigenetic changes through AMPK-dependent phosphorylation of HDAC5, facilitating its export from the nucleus and relieving repression of the PGC-1α promoter [67]. Furthermore, exercise-induced irisin activates AMPK signaling in renal tubular epithelial cells, creating a positive feedback loop that enhances mitochondrial protection and biogenesis in the kidney [12]. The metabolic and mitochondrial adaptations observed in this study are further supported by recent research. One study showed that exercise training restores mitochondrial metabolic flexibility and lipid oxidation capacity in obese experimental models, aligning with the improvements in citrate synthase activity, Complex I function, and ATP levels observed in Group IV [49]. Han et al. [11] demonstrated that irisin exerts protective effects against obesity-related CKD by modulating perirenal adipose tissue function and the VEGF–NO axis in renal glomeruli, providing an independent experimental framework that supports the irisin-mediated renoprotective mechanisms proposed here. Collectively, these findings strengthen the importance of the irisin–AMPK–PGC-1α axis as a promising therapeutic target for obesity-related kidney injury. We acknowledge that our study is associative and lacks rescue experiments (e.g., FNDC5 knockdown, AMPK inhibition, exogenous irisin) to establish strict causality. However, convergent multi-level evidence, including biochemical, molecular, mitochondrial, and histopathological data, consistently aligns with the irisin–AMPK–PGC-1α axis and is independently corroborated by published interventional studies (Formigari et al. [12]; Lu et al. [57]; Han et al. [11]). The controlled four-group design systematically isolates the effects of obesity and exercise. While definitive causal proof awaits future interventional studies, the biological plausibility and internal consistency of our findings support the conclusions.

### Study Limitations

Several limitations should be acknowledged:

1. The design is associative rather than causal; mechanistic rescue experiments, including FNDC5/irisin knockdown, pharmacological AMPK inhibition (e.g., dorsomorphin), and exogenous irisin supplementation, are required to confirm that the observed renoprotective effects are mechanistically dependent on the irisin–AMPK–PGC-1α axis.
2. Immunofluorescence co-localization of phospho-AMPKα1, PGC-1α, and integrin αV/β5 in renal tubular cells was not performed and would have strengthened the mechanistic link to the irisin receptor.
3. Only one exercise modality (swimming) was evaluated, limiting generalizability.

## 5. Conclusions

This study provides convergent biochemical, molecular, and histopathological evidence that swimming exercise protects the kidneys from obesity-induced injury via an irisin–AMPK–PGC-1α axis that restores mitochondrial energy production and reduces renal cell death. These findings identify irisin as a key exercise-related factor linking skeletal muscle metabolic health to kidney protection and underscore the AMPK–PGC-1α pathway as an important therapeutic target in obesity-related nephropathy. Although strict causality cannot be established without rescue experiments, the consistency of evidence across multiple levels supports irisin as a candidate exercise-derived mediator and the AMPK–PGC-1α pathway as a potential therapeutic target. Future studies using irisin supplementation, AMPK inhibition, or gene-specific knockdown approaches are essential to confirm causal relationships and explore clinical translation.

## Figures and Tables

**Table 1:** Body weight and kidney weight across the four study groups.

Parameter	Group I	Group II	Group III	Group IV	Statistic	*p*-Value
Initial BW (g) §	117.13 ± 5.50	118.38 ± 4.46	114.30 ± 3.91	116.67 ± 4.81	F = 1.97	0.129
BW at 12 weeks (g) ‡	176.10 ± 3.71	295.80 ± 9.71 ^a^	170.20 ± 3.01 ^a,b^	315.57 ± 7.68 ^a,b,c^	F = 2121.65	**<0.001**
Final BW (g) † After 8 weeks of exercise training	252.59 [248.21, 256.36]	345.65 [335.59, 352.21] ^a^	248.69 [238.92, 254.36] ^b^	272.31 [269.10, 274.86] ^a,b,c^	H = 44.04	**<0.001**
Kidney weight (g) †	0.79 [0.76, 0.81]	2.24 [2.13, 2.27] ^a^	0.67 [0.63, 0.75] ^b^	1.61 [1.52, 1.91] ^a,c^	H = 51.19	**<0.001**

Data are expressed as mean ± SD or median [Q1, Q3]. § One-way ANOVA with Tukey’s HSD post hoc test; ‡ Welch’s ANOVA with Games–Howell post hoc test; † Kruskal–Wallis test with Dunn’s post hoc test (Bonferroni-adjusted). Superscripts indicate significant differences: a vs. Group I; b vs. Group II; c vs. Group III (*p* < 0.05). BW, body weight. Bold *p*-values indicate statistical significance (*p* < 0.05). n = 15 per group.

**Table 2:** Renal function tests, lipid profiles, and glycemic parameters across the four study groups.

Parameter	Group I	Group II	Group III	Group IV	Statistic	*p*-Value
Urea (mg/dL) ‡	20.16 ± 1.14	38.31 ± 0.87 ^a^	21.56 ± 1.87 ^b^	23.10 ± 1.94 ^a,b^	F = 948.7	**<0.001**
Creatinine (mg/dL) ‡	0.39 ± 0.07	0.79 ± 0.21 ^a^	0.44 ± 0.03 ^b^	0.57 ± 0.09 ^a,b,c^	F = 25.88	**<0.001**
U-Albumin (mg/24 h) †	10.71 [10.48, 10.98]	34.27 [33.22, 34.52] ^a^	10.73 [10.39, 11.57] ^b^	19.77 [19.44, 19.96] ^a,c^	H = 49.83	**<0.001**
Triglycerides (mg/dL) ‡	82.13 ± 2.88	329.55 ± 10.10 ^a^	86.12 ± 5.07 ^b^	214.98 ± 6.69 ^a,b,c^	F = 3988.9	**<0.001**
Total cholesterol (mg/dL) ‡	80.69 ± 2.62	309.46 ± 8.50 ^a^	83.95 ± 1.02 ^a,b^	167.51 ± 10.25 ^a,b,c^	F = 3618.9	**<0.001**
LDL-C (mg/dL) †	26.59 [24.83, 27.70]	224.60 [220.23, 231.49] ^a^	28.23 [26.14, 30.46] ^b^	94.10 [90.96, 96.52] ^a,c^	H = 50.57	**<0.001**
HDL-C (mg/dL) §	35.13 ± 2.68	24.16 ± 3.78 ^a^	34.62 ± 3.73 ^b^	30.93 ± 1.88 ^a,b,c^	F = 39.40	**<0.001**
Fasting glucose (mg/dL) §	84.90 ± 7.62	138.41 ± 6.76 ^a^	85.75 ± 5.99 ^b^	110.62 ± 4.18 ^a,b,c^	F = 244.50	**<0.001**
Fasting insulin (µIU/L) ‡	5.09 ± 0.70	34.03 ± 3.56 ^a^	5.42 ± 0.94 ^b^	11.91 ± 0.90 ^a,b,c^	F = 460.3	**<0.001**
HOMA-IR †	1.07 [0.98, 1.13]	11.41 [10.53, 11.68] ^a^	1.06 [0.98, 1.09] ^b^	3.22 [2.89, 4.03] ^a,c^	H = 49.91	**<0.001**

Data are expressed as mean ± SD or median [Q1, Q3] depending on distribution. † Kruskal–Wallis with Dunn’s post hoc test (Bonferroni-adjusted); ‡ Welch’s ANOVA with Games–Howell post hoc test; § One-way ANOVA with Tukey’s HSD post hoc test. Superscripts indicate significant differences: a vs. Group I; b vs. Group II; c vs. Group III (*p* < 0.05). U-Albumin, urinary albumin; LDL-C, low-density lipoprotein cholesterol; HDL-C, high-density lipoprotein cholesterol; HOMA-IR, homeostasis model assessment of insulin resistance. Bold *p*-values indicate statistical significance. n = 15 per group.

**Table 3:** Irisin levels, PGC-1α protein, and citrate synthase activity in muscle tissue across the four study groups.

Parameter	Group I	Group II	Group III	Group IV	Statistic	*p*-Value
Irisin (ng/mg ptn) †	2.08 [1.94, 2.34]	0.74 [0.57, 0.98] ^a^	3.33 [2.42, 3.56] ^b^	1.65 [1.13, 1.80] ^c^	H = 48.26	**<0.001**
PGC-1α (ng/mg ptn) †	1.89 [1.82, 1.99]	0.78 [0.64, 0.86] ^a^	2.60 [1.95, 3.02] ^b^	1.48 [1.08, 1.74] ^b^	H = 36.92	**<0.001**
CS activity (nmol/min/mg ptn) ‡	10.97 ± 2.61	2.86 ± 1.11 ^a^	14.73 ± 2.62 ^a,b^	8.02 ± 3.50 ^b,c^	F = 109.3	**<0.001**

Data are expressed as median [Q1, Q3] or mean ± SD. † Kruskal–Wallis with Dunn’s post hoc test; ‡ Welch’s ANOVA with Games–Howell post hoc test. Superscripts indicate significant differences: a vs. Group I; b vs. Group II; c vs. Group III (*p* < 0.05). CS, citrate synthase; ptn, protein. Bold *p*-values indicate statistical significance. n = 15 per group.

**Table 4:** Renal mitochondrial function markers, PGC-1α mRNA expression, and oxidative stress markers across the four study groups.

Parameter	Group I	Group II	Group III	Group IV	Statistic	*p*-Value
p-AMPKα1(pg/µg ptn) †	0.09 [0.08, 0.09]	0.06 [0.05, 0.06] ^a^	0.09 [0.09, 0.10] ^b^	0.08 [0.07, 0.08] ^c^	H = 46.56	**<0.001**
Complex I (µmol NADH/min/mg ptn) ‡	88.64 ± 14.38	38.39 ± 16.32 ^a^	106.07 ± 8.39 ^a,b^	68.09 ± 19.24 ^a,b,c^	F = 71.45	**<0.001**
ATP (µmol/mg ptn) §	0.99 ± 0.11	0.52 ± 0.08 ^a^	1.15 ± 0.11 ^a, b^	0.86 ± 0.09 ^a, b,c^	F = 111.3	**<0.001**
PGC-1α mRNA (RE) †	1.95 [1.90, 2.00]	0.69 [0.60, 0.74] ^a^	2.27 [2.17, 2.62] ^b^	1.04 [1.00, 1.06] ^a,c^	H = 51.52	**<0.001**
Renal MDA-Level (nmol/mg Protein) §	1.17 ± 0.21	3.96 ± 0.29 ^a^	1.12 ± 0.21 ^b^	1.98 ± 0.25 ^a,b,c^	F =405.07	**<0.001**
SOD (Units/mg Protein) §	18.9 ± 0.73	5.98 ± 0.53 ^a^	18.34 ± 0.79 ^b^	14.33 ± 1.2 ^a,b,c^	F =695.98	**<0.001**

Data are expressed as median [Q1, Q3] or mean ± SD. † Kruskal–Wallis with Dunn’s post hoc test; ‡ Welch’s ANOVA with Games–Howell post hoc test; § One-way ANOVA with Tukey’s HSD post hoc test. Superscripts indicate significant differences: a vs. Group I; b vs. Group II; c vs. Group III (*p* < 0.05). p-AMPKα1, phosphorylated AMP-activated protein kinase alpha-1; RE, relative expression; ptn, protein. Bold *p*-values indicate statistical significance. n = 15 per group.

**Table 5:** Semi-quantitative histological scoring of renal tissue alterations across experimental groups.

Parameter	Group I (Control Non-Trained)	Group II (Obese Non-Trained)	Group III (Control Trained)	Group IV (Obese Trained)
**Renal corpuscle**				
Glomerulosclerosis	0.00 ± 0.00 ^a^	1.83 ± 0.75 ^c^	0.00 ± 0.00 ^a^	0.83 ± 0.75 ^b^
Glomerular necrosis	0.00 ± 0.00 ^a^	1.83 ± 0.41 ^c^	0.00 ± 0.00 ^a^	0.83 ± 0.75 ^b^
Bowman’s space dilation	0.00 ± 0.00 ^a^	2.33 ± 0.52 ^c^	0.00 ± 0.00 ^a^	1.00 ± 0.63 ^b^
**Renal tubules**				
Dilation	0.00 ± 0.00 ^a^	2.17 ± 0.75 ^c^	0.00 ± 0.00 ^a^	1.00 ± 0.63 ^b^
Coagulative necrosis	0.00 ± 0.00 ^a^	2.00 ± 0.63 ^c^	0.00 ± 0.00 ^a^	0.83 ± 0.75 ^b^
Cloudy swelling	0.00 ± 0.00 ^a^	2.67 ± 0.52 ^c^	0.00 ± 0.00 ^a^	1.17 ± 0.41 ^b^
Fat droplet deposition	0.00 ± 0.00 ^a^	2.50 ± 0.55 ^c^	0.00 ± 0.00 ^a^	1.00 ± 0.63 ^b^
**Interstitium**				
Congestion	0.00 ± 0.00 ^a^	2.17 ± 0.75 ^c^	0.00 ± 0.00 ^a^	1.17 ± 0.41 ^b^
Inflammatory infiltration	0.00 ± 0.00 ^a^	2.50 ± 0.55 ^c^	0.00 ± 0.00 ^a^	1.00 ± 0.63 ^b^

Data are expressed as mean ± SD. Histopathological lesions were scored on a scale of 0–3 (0 = no lesion, 1 = mild, 2 = moderate, 3 = severe). Different letters (a, b, c) indicate statistically significant differences between groups at *p* < 0.05, determined by one-way ANOVA followed by Tukey’s post hoc test.

## Data Availability

The data supporting the findings of this study are included in the manuscript.

## Abbreviations

The following abbreviations are used in this manuscript:

AMPK	Adenosine Monophosphate-Activated Protein Kinase
ATP	Adenosine Triphosphate
BSA	Bovine Serum Albumin
CKD	Chronic Kidney Disease
CS	Citrate Synthase
DAB	3,3′-Diaminobenzidine
DTNB	5,5′-Dithiobis (2-nitrobenzoate)
EDTA	Ethylenediaminetetraacetic Acid
ELISA	Enzyme-Linked Immunosorbent Assay
FFA	Free Fatty Acids
FNDC5	Fibronectin Type III Domain-Containing Protein 5
H&E	Hematoxylin and Eosin
HDL-C	High-Density Lipoprotein Cholesterol
HFD	High-Fat Diet
HOMA-IR	Homeostatic Model Assessment for Insulin Resistance
LDL-C	Low-Density Lipoprotein Cholesterol
OAA	Oxaloacetate
PBS	Phosphate-Buffered Saline
PGC-1α	Peroxisome Proliferator-Activated Receptor Gamma Coactivator-1α
qRT-PCR	Quantitative Real-Time Polymerase Chain Reaction
TC	Total Cholesterol
TG/TAG	Triglycerides
UAER	Urinary Albumin Excretion Rate
